# Cytokine & chemokine response in the lungs, pleural fluid and serum in thoracic surgery using one-lung ventilation

**DOI:** 10.1186/1476-9255-8-32

**Published:** 2011-11-11

**Authors:** Andreas Breunig, Franco Gambazzi, Beatrice Beck-Schimmer, Michael Tamm, Didier Lardinois, Daniel Oertli, Urs Zingg

**Affiliations:** 1 Department of Surgery, University Hospital Basel, Switzerland; 2 Thoracic Surgery, University Hospital Basel, Switzerland; 3 Institute of Anaesthesiology, University Hospital Zürich, Switzerland; 4 Pulmonary Medicine, University Hospital Basel, Switzerland

**Keywords:** Inflammation, cytokines, chemokines, thoracic surgery, one-lung ventilation

## Abstract

**Background:**

Thoracic surgery mandates usually a one-lung ventilation (OLV) strategy with the collapse of the operated lung and ventilation of the non-operated lung. These procedures trigger a substantial inflammatory response. The aim of this study was to analyze the cytokine and chemokine reaction in both lungs, pleural space and blood in patients undergoing lung resection with OLV with special interest in the chemokine growth-regulated peptide alpha (GROα) which is the human equivalent to the rat cytokine-induced neutrophil chemoattractant-1 (CINC-1).

**Methods:**

Broncho-alveolar lavage (BAL) fluid of both the collapsed, operated and the ventilated, non-operated lung, respectively, pleural space drainage fluid and blood was collected and the concentrations of interleukin (IL)-6, IL-1RA and GROα were determined with enzyme-linked immunosorbent assays in 15 patients.

**Results:**

Substantial inter-individual differences in the BAL fluid between patients in cytokine and chemokine levels occurred. In the pleural fluid and the blood these inter-individual differences were less pronounced. Both sides of the lung were affected and showed a significant increase in IL-6 and IL-1RA concentrations over time but not in GROα concentrations. Except for IL-6, which increased more in the collapsed, operated lung, no difference between the collapsed, operated and the ventilated, non-operated lung occurred. In the blood, IL-6 and IL-1RA increased early, already at the end of surgery. GROα was not detectable. In the pleural fluid, both cytokine and chemokine concentrations increased by day one. The increase was significantly higher in the pleural fluid compared to the blood.

**Conclusion:**

The inflammatory response of cytokines affects both the collapsed, operated and the ventilated, non-operated lungs. The difference in extent of response underlines the complexity of the inflammatory processes during OLV. In contrast to the cytokines, the chemokine GROα concentrations did not react in the BAL fluid or in the blood. This indicates that GROα might not be useful as marker for the inflammatory reaction in complex surgical procedures.

## Background

Thoracic surgery such as esophagectomy or lobectomy triggers a more severe systemic inflammatory reaction than intra-abdominal surgery [1]. One possible explanation is the fact that most thoracic procedures mandate a one-lung ventilation (OLV) strategy. The OLV leads to a collapse of the lung that is operated with subsequent shunting of blood and possible hypoxemia. The contralateral lung is ventilated and may suffer from ventilator-induced injury, hyperoxia and increased capillary stress [2-4].

A number of studies investigated cytokine levels in thoracic surgery [5-9]. The degree of inflammatory response depended on the extent of the surgical trauma (thoracotomy vs. video-assisted thoracic surgery) and the nature of the disease (benign vs. malign) [5-7]. Cytokine levels, especially interleukin-6 (IL-6) and interleukin-8 (IL-8), were associated with postoperative infections and systemic inflammatory response syndrome, which itself was a predictive factor the duration of the hospital stay [8,9]. The influence of the OLV on the inflammatory process is still unclear. Most studies addressing cytokine production in thoracic surgery measured serum cytokine levels. Measurements in other compartments such as the pleural space and the collapsed and ventilated lungs by bronchoalveolar lavage (BAL) are scarce. Cree et al. demonstrated higher IL-8 levels in the BAL fluid after esophagectomy compared to the peripheral blood [10]. These authors also analyzed BAL samples from both lungs without showing a difference. However, the authors compared right and left lungs of two different groups of patients. Our group demonstrated a more pronounced inflammatory response on the ventilated left lung in patients undergoing transthoracic esophagectomy [11]. As cytokine concentrations vary substantially between patients, it is important to analyze both lungs of the same patient.

A variety of chemokines have recently been shown to play an important role in inflammation. One is the rat cytokine-induced neutrophil chemoattractant-1 (CINC-1). CINC-1 is an acute phase protein with the ability to recruit neutrophils [12]. The human equivalent to the rat CINC-1 is the growth-regulated peptide alpha GROα. To our knowledge, the role of GROα as human equivalent to CINC-1 in the inflammatory reaction in patients undergoing thoracic surgery has not been studied so far.

Thus, the aim of this study was to analyze the inflammatory response represented by the pro-inflammatory cytokine IL-6, the anti-inflammatory cytokine interleukin-1 receptor antagonist (IL-1RA) and the chemokine GROα in the operated, collapsed and the non-operated, ventilated lungs, in the pleural space and the peripheral blood in patients undergoing lobectomy for cancer with special interest in the reaction of the chemokine GROα. We hypothesized that the pro-inflammatory GROα shows a similar reaction to the pro-inflammatory cytokine IL-6.

## Patients and methods

From the 1^st ^of June 2007 all patients undergoing thoracotomy and resection for non-small cell lung cancer at the University Hospital Basel, Switzerland, were assessed for eligibility to be included in this prospective observational study. Exclusion criteria were pneumonectomy, minimal invasive procedures and refusal of the patient to participate. The Ethics committee of the state of Basel has approved the study. Informed consent was obtained from all patients.

### Anesthetic procedure and surgery

Anesthetic procedures were standardized for all patients including the use of double-lumen endobronchial tubes under fiber-optic control to allow single lung ventilation. All patients received a thoracic epidural catheter with continuous infusion of local anesthetics both intraoperatively (ropivacaine 0.3%) and postoperatively (bupivacaine 0.125%). Total intravenous anesthesia was applied using bolus doses of fentanyl and continuous infusions of propofol and remifentanil intraoperatively and tempered until endotracheal extubation. Muscle relaxation (intraoperatively only) was achieved using bolus doses of rocuronium. One-lung ventilation during thoracotomy followed the principles of a lung protective strategy, using pressure-limited or pressure-controlled ventilation modes with tidal volumes of <7 ml/kg body weight, positive end-expiratory pressure of 3-5 cm H2O and limiting peak inspiratory pressures to <30 cm H2O. Inspired oxygen concentration on the ventilated lung was set to 100% at the beginning and gradually reduced thereafter based on the arterial oxygen tension measured by repeated blood gas analyses [13-15]. Continuous monitoring included ECG analysis, measurement of arterial oxygen saturation with pulse oxymetry and invasive arterial as well as central venous pressure monitoring. Blood samples were drawn intermittently at predefined time points for blood gas and further laboratory parameter analysis.

Surgery was performed by two consultant thoracic surgeons with the following surgical technique: after performing a lateral thoracotomy with entering the pleural cavity either through the fourth or the fifth intercostal space the lobe with the tumor was mobilized by preparation of the hilum and the interlobar fissures. During this procedure a part of the systematic mediastinal lymph node dissection according to the American Thoracic Society mapping was performed [16]. Then the tumor was resected either by a lobectomy, a bilobectomy, a sleeve-lobectomy or by an anatomical segmentectomy using staplers for the vascular, bronchial and parenchymal resection. After completing the systematic lymph node dissection the operation was finished by positioning one or two chest tubes.

### BAL, pleural lavage and peripheral blood samples

Bilateral BAL was performed in all patients in supine position after intubation and at the completion of surgery. The bronchoscope was wedged in the lower bronchus of both lungs and a lavage with 50ml of sterile saline was performed. The BAL fluid was immediately centrifuged at 2500rpm for 15 minutes and the supernatant stored at -20°C.

Lavage of the pleural space was performed after thoracotomy and before closure of the thoracotomy with 100ml of sterile saline. On day 1-3, pleural fluid samples were taken in a standardized way. The tubes were clamped close to the chest and the collection system. Then the pleural fluid was aspirated from the tube using a standard 20 gauge needle. All samples were taken by the same investigator. The fluid was processed as described above for the BAL samples.

Peripheral venous blood samples were obtained at the same time as the BAL and pleural fluid samples on the day of surgery and on day 1-3. Again, the samples were processed as described.

### Cytokine assays

The concentrations of IL-6, IL-1RA and GROα in the BAL, pleural space and blood samples were determined with enzyme-linked immune-sorbent assays (ELISA, R & D Systems, Minneapolis, MN, USA). To standardize the BAL fluid samples for optimal comparison, we extrapolated the results retrieved in the ELISA to a sample volume of 10ml.

### Statistical analysis

Comparison of data between the groups was undertaken using Chi-square tests for categorical data, and Wilcoxon signed rank tests for continuous data. Data is presented as mean values with standard deviation (SD) or median values with inter-quartile range (IQR) as appropriate. Based on a paired t-test with a type 1 error set at 0.05 the sample size of 15 patients allowed an 82% power to detect a size effect difference of 0.8. Statistical significance was set at p < 0.05. Statistical analyses were performed with Medcalc^®^, Version 9 for Windows.

## Results

### Patients, pathology and morbidity

There were 7 men and 8 women in the study cohort. The mean age was 65 years (SD 13 years) and mean body mass index was 24.9 kg/m^2 ^(SD 5.8 kg/m^2^). Preoperative pulmonary function tests showed a mean forced expiratory volume (FEV1) of 2.3 liters (SD 0.9 liters) and a vital capacity (VC) of 3.3 liters (SD 1.1 liters). The mean FEV1/VC percentage was 71.0% (SD 14.1.0%). Mean cardiac ejection fraction determined by preoperative echocardiography was 56.5% (SD 7.0%).

Chronic obstructive pulmonary disease was present in three patients (20%), five patients had cardiac comorbidities (33%, two with atrial fibrillation, three with coronary heart disease), and five patients (33%) had pre-existing renal impairment. Table 1 summarizes the surgical, anesthetic and the pathological data. There were no deaths and no surgical morbidity. The only postoperative morbidity was two patients with pneumonia.

**Table 1:** Surgical and pathological data

SURGICAL PROCEDURES	
LOBECTOMY	11
BI-LOBECTOMY	1
SEGMENTECTOMY	1
SLEEVE LOBECTOMY	2
MEAN LENGTH OF OPERATION IN MINUTES (SD)	190 (59)

MEAN LENGTH OF ANAESTHETIC PROCEDURE IN MINUTES (SD)	402 (91)

MEAN LENGTH OF ONE LUNG VENTILATION IN MINUTES (SD)	156 (73)

MEAN BLOOD LOSS IN MILILITRES (SD)	420 (156)

TYPE OF TUMOR	
LARGE CELL CARCINOMA	3
ADENOCARCINOMA	9
SQUAMOUS CELL CANCER	3

### BAL fluid cytokine assay

**Figure 1:**
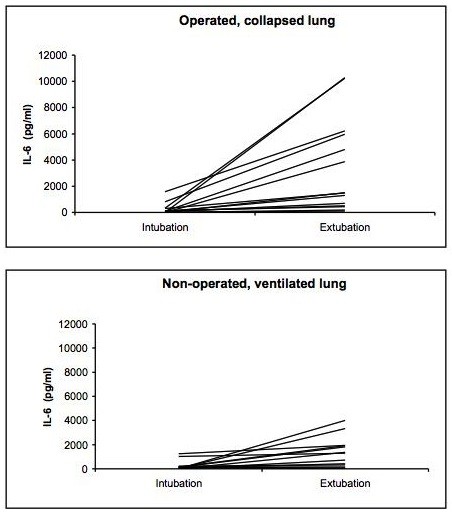
**Course of IL-6 in the operated collapsed and non-operated ventilated lungs in the individual 15 patients**.

**Figure 2:**
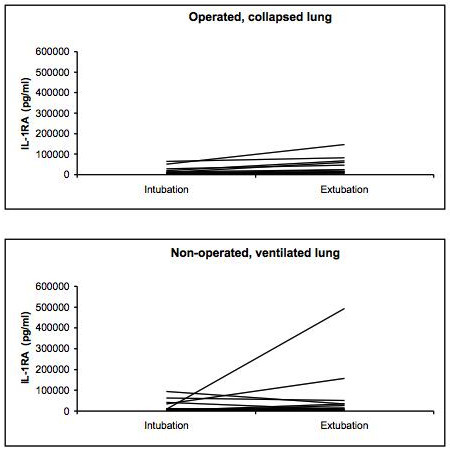
**Course of IL-1RA in the operated collapsed and non-operated ventilated lungs in the individual 15 patients**.

**Figure 3:**
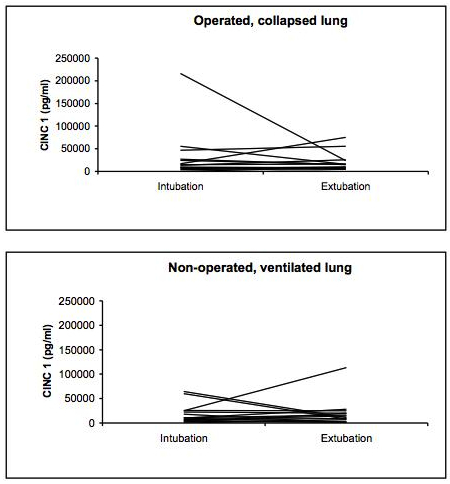
**Course of CINC-1 in the operated collapsed and non-operated ventilated lungs in the individual 15 patients**.

The BAL cytokine and chemokine measurements showed substantial inter-individual differences in both the operated, collapsed and non-operated, ventilated lung for all mediators as demonstrated for IL-6 as example in Figures 1, 2 and 3. The results of the BAL measurements are demonstrated in table 2. Except for IL-6, no differences between the operated, collapsed and non-operated, ventilated lung occurred. Over time (at intubation vs. end of surgery) the pro-inflammatory IL-6 and the anti-inflammatory IL-1RA showed a significant increase in the operated, collapsed lung (IL-6 p < 0.001, IL-1RA p < 0.001), whereas in the non-operated, ventilated lung only IL-6 significantly increased (p = 0.006) but not IL-1RA (p = 0.525). GROα showed no significant change over time (operated, collapsed lung p = 0.720, non-operated, ventilated lung p = 0.978).

**Table 2:** Comparison of the median and mean cytokine levels in the BAL fluid of the right and left lung

	BAL COLLAPSED OPERATED LUNG	RATIO OF CYTOKINE RESPONSE COLLAPSED OPERATED LUNG*	BAL VENTILATED NON-OPERATED LUNG	RATIO OF CYTOKINE RESPONSE VENTILATED NON-OPERATED LUNG*	P VALUE# BAL OPERATED COLLAPSED LUNG COMPARED TO BAL NON-OPERATED VENTILATED LUNG
IL-6 (PG/ML), MEDIAN/MEAN (IQR)					
AT INTUBATION	63/236 (4-271)	13.5	118/222 (12-148)	5.2	1.000
END OF SURGERY	1486/3176 (452-5681)		710/1159 (99-1899)		0.010

IL-1RA (PG/ML), MEDIAN/MEAN (IQR)					
AT INTUBATION	7144/15289 (3316-17921)	2.3	7144/19863 (3469-29394)	2.9	0.670
END OF SURGERY	14837/35242 (9803-56578)		12878/57543 (5943-35014)		0.252

GROα (PG/ML), MEDIAN/MEAN (IQR)					
AT INTUBATION	13467/30597 (6967-26499)	0.6	10137/18043 (5132-24379)	1.0	0.720
END OF SURGERY	15133/19187 (7330-22047)		11252/18552 (3487-20038)		0.599

*Concentration at intubation as base line for calculation of ratio

# Wilcoxon paired samples test

### Blood and pleural fluid cytokine assays

**Figure 4:**
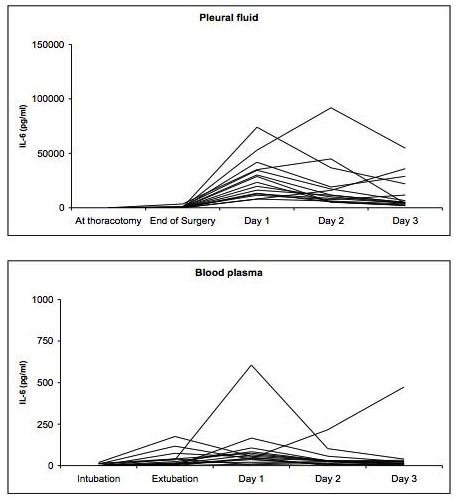
**Course of IL-6 in the pleural fluid and blood plasma in the individual 15 patients**.

The pleural fluid and blood cytokine assays demonstrated less inter-individual differences as shown for IL-6 in Figure 4. IL-1RA demonstrated a similar inter-individual pattern whereas GROα did not. Table 3 shows the results of the pleural and blood cytokine and chemokine assays. The cytokines reacted significantly more pronounced in the pleural fluid compared to the blood, starting with a certain delay on the first postoperative day.

**Table 3:** Comparison of the median and mean cytokine levels in the pleura fluid with the levels in the peripheral blood.

	PLEURAL FLUID	RATIO OF CYTOKINE RESPONSE PLEURAL FLUID*	PERIPHERAL BLOOD	RATIO OF CYTOKINE RESPONSE PERIPHERAL BLOOD*	P VALUE# PLEURAL FLUID VERSUS BLOOD
IL-6 (PG/ML), MEDIAN/MEAN (IQR)					
AT THORACOTOMY/INTUBATION	0/14 (0-21)	29.8	0/4 (0-10)	9.5	0.109
END OF SURGERY	58/417 (22-396)	1950.6	16/38 (7-41)	24.8	0.085
DAY 1	23448/27309 (12900-34929)	1889.9	54/99 (39-84)	10.5	<0.001
DAY 2	11396/26458 (6510-18582)	914.3	28/42 (16-31)	11.0	<0.001
DAY 3	4709/12800 (3590-19392)		13/44 (2-24)		<0.001

IL-1RA (PG/ML), MEDIAN/MEAN (IQR)					
AT THORACOTOMY/INTUBATION	9/46 (0-76)	5.8	75/470 (0-979)	1.2	0.102
END OF SURGERY	140/267 (11-252)	440.8	134/580 (0-913)	1.6	0.525
DAY 1	16239/20277 (10210-23527)	335.5	516/735 (0-1251)	1.1	<0.001
DAY 2	8062/15435 (3477-15589)	261.3	112/492 (4-949)	1.1	<0.001
DAY 3	4364/12018 (3152-13764)		147/524 (0-1184		<0.001

GROα (PG/ML), MEDIAN/MEAN (IQR)					
AT THORACOTOMY/INTUBATION	3/17 (0-30)	2.9	0/183 (0-257)	1.1	0.193
END OF SURGERY	25/50 (7-57)	15.2	0/201 (0-224)	0.7	0.525
DAY 1	172/258 (118-306)	11.2	0/136 (0-282)	0.6	0.017
DAY 2	64/190 (25-179)	7.4	0/114 (0-145)	0.6	0.268
DAY 3	97/125 (29-182)		0/105 (0-116)		0.389

The pleural samples were taken immediately after the thoracotomy and before closure of the thoracotomy. On day 1-3 the pleural and peripheral blood samples were taken at the same time.

*Concentration at intubation as base line for calculation of ratio (means used for calculation)

# Wilcoxon paired samples test

The pro-inflammatory IL-6 significantly increased early in the blood, already at the end of surgery, thus only a few hours after the surgical trauma (at intubation 4 pg/ml, at end of surgery 38 pg/ml; p = 0.001). From the end of surgery to day one, the concentrations increased further, however, not reaching statistical significance (at end of surgery 38 pg/ml, day one 99 pg/ml; p = 0.153). Although the anti-inflammatory IL-1RA also increased, this increase was not significant. GROα was not detectable in the blood.

In the pleural fluid, IL-6 levels increased on day one, but not at the end of surgery (at intubation 14 pg/ml, at the end of surgery 417 pg/ml; p = 0.168; day one 27309 pg/ml; p < 0.001). IL-1RA levels were significantly higher at the end of surgery compared to the baseline at the intubation, and increased more significantly on day one (at intubation 46 pg/ml, at the end of surgery 267 pg/ml; p = 0.034; day one 20277 pg/ml; p < 0.001). GROα showed a similar pattern as IL-1RA (at intubation 17 pg/ml, at the end of surgery 50 pg/ml; p = 0.007; day one 258 pg/ml; p < 0.001).

## Discussion

The main finding of this study is the fact that the chemokine GROα did not react similarly to the cytokines IL-6 and IL-1RA in the BAL and the blood. All patients showed a significant increase of the bronchoalveolar cytokines in both the operated, collapsed and non-operated, ventilated lungs. Analyzing BAL fluid from both lungs of the same patient is important to avoid bias due to the substantial variation in cytokine levels in the individual patient [10,17]. Our results support this finding as the inter-individual difference in both the operated, collapsed and the non-operated, ventilated lung, respectively, was substantial for all three mediators. To our knowledge, no study has analyzed the inflammatory response in both lungs of the same patient in open lung surgery. In most patients the increase of IL-6 levels in the BAL fluid was moderate, but in some patients the IL-6 levels were very high, explaining the differences between the median and mean values. In contrast, in the pleural fluid and the blood the inter-patient variability was less pronounced for IL-6 and IL-1RA, and no variability occurred for GROα. Some patients had a more pronounced reaction compared with the majority, both in the pleural fluid and in the blood. The reason for this remains unclear, as we did not detect any difference in the frequency of inflammatory complications between these patients. A number of studies reported an inter-subject variability of various inflammatory markers, but again the exact reasons remain unclear [18,19]. Aaron et al. analyzed sputum and serum specimens from patients with stable chronic obstructive pulmonary disease and controls, and found similar results to our study, i.e. a more substantial reaction in the specimen sampled directly from the lungs [19]. These authors explained their finding with the higher variability in day-to-day amount and dilution of analyzed sputum, however, after correction of their results by normalizing for the sputum total protein some of the variability remained.

The pro-inflammatory IL-6 reacted more on the operated, collapsed lung and showed in median a 13-time higher concentration in the BAL fluid at the end of surgery. The anti-inflammatory IL-1RA reacted in both lungs similarly, with a two- to three-fold increase. This is in contrast to a recent analysis of our group in patients who underwent transthoracic esophagectomy with OLV, where a more pronounced reaction on the ventilated side was observed [11]. One possible explanation for this difference is the fact that in lung surgery the tissue damage is created directly on the lung, thus triggering a more severe reaction on the operated side that is subsequently measured in the BAL fluid. In contrast, in esophagectomies the inflammatory response is likely to be triggered by the OLV itself through high oxygen concentrations and the direct mechanical stress on the alveolar walls [20].

GROα showed no increase in the BAL fluid. GROα is the human equivalent to the rat CINC-1 that is an acute phase protein and induces recruitment of neutrophils, thus is a pro-inflammatory mediator. We hypothesized that GROα levels would increase similar to the pro-inflammatory cytokines, in both lungs. Only limited data on chemokines after surgical procedures exist. Seitz et al. showed that chemokine levels in BAL fluid of rats subjected to blunt chest trauma increased compared to sham [21]. Schilling et al. demonstrated an increase of intra-alveolar granulocytes in the ventilated lung in patients undergoing thoracic surgery with OLV [22]. In their study, the increase in cells occurred two hours postoperatively. One reason for the lack in increase of GROα levels in our study might be the time course of the chemokine in the lung. In our study, GROα levels peaked in the pleural fluid on day one whereas no substantial increase was seen at the end of surgery. This might indicate a slower increase over time. The role of the various chemokines in inflammation is not clear, however, their potential role in targeting and blocking inflammatory processes has been recognized in hepatic diseases, rheumatoid arthritis or allergic asthma [23,24].

In the peripheral blood, the pro-inflammatory IL-6 levels increased substantially and peaked on day one. The anti-inflammatory IL-1RA showed also an increase, but less pronounced. Similar findings have been described in most major surgical procedures. However, transthoracic procedures seem to trigger a more pronounced pro-inflammatory reaction [1]. The main reason seems to be the thoracotomy. Yim et al. demonstrated lower IL-6 levels in video-assisted thoracic surgery (VATS) compared to open thoracic surgery [5]. For both, IL-6 and IL-1RA, the increase in the peripheral blood occurred already at the end of surgery, thus early in the time course and only shortly after the surgical trauma occurred. Also, both cytokines showed significantly higher levels in the locally sampled fluids, i.e. BAL and pleural fluid. The inflammatory reaction seems to be generated primarily locally. Also, depression of cytokine production in the peripheral blood might contribute to this less pronounced increase [25]. Considering the high postoperative morbidity of these operations, the early postoperative IL-6 levels could be of interest for early detection of infectious complications. IL-6 has been detected as early marker for postoperative sepsis [26]. In our study, no analysis stratified according postoperative morbidity was possible due to a low number of patients and a very low postoperative complication rate.

Similar to the levels in the BAL fluid, we were not able to detect an increase in the GROα levels in the blood. On postoperative day one, the GROα level in the blood was even decreased. We were not able to explain this finding. Our results indicate that the usefulness of GROα levels in the peripheral blood as markers of inflammation may be limited.

In the pleural fluid, IL-6, IL-1RA and GROα increased. Similar to the blood, the peak occurred on day one, but the increase was already detectable at the end of the surgery. The levels in the pleural fluid were significantly higher compared to the blood. This finding is consistent to other reports measuring pleural fluid cytokine levels in patients undergoing esophagectomy [27,28]. Overall, data on the inflammatory reaction in the pleural space is scarce. Weissflog et al. demonstrated higher pleural concentrations of IL-10 and other cytokines in non-malignant diseases, and in patients undergoing VATS [29]. Szczesny et al. showed substantially higher levels of IL-6 and IL-1RA in the pleural fluid compared to the serum [30]. The elevation of cytokines persists to day three, which may also be influenced by the thoracic drains. The pleural cavity seems to be an important location of inflammation. GROα levels were only elevated in this compartment. GROα is a potent chemoattractant for neutrophils, and this influx of cells might also influence the tumor-immunological environment. The production of oxygen radicals by neutrophils might decrease the ability of anti-tumor acting cells, such as natural killer cells, to eliminate free tumor cells.

The main limitation of this study is the number of patients, and thus, our results mandate careful interpretation. However, with our sample size of 15 patients we were powered up to detect a major difference between operated and non-operated lungs.

Performing BAL on both lungs of each patients is complex, time consuming and not without risk. The results presented in this study are, to our knowledge, the first demonstrating the time course of chemokines in lung surgery and give a first insight in the cytokine and chemokine reaction in complex thoracic surgery. Further studies to determine the impact of the loco-regional inflammatory response and its clinical relevance after thoracic surgery are necessary.

## Conclusion

The inflammatory response of pro- and anti-inflammatory cytokines affects both the collapsed operated and the ventilated contralateral lungs. The inter-individual difference was present in the BAL of the lungs but less in the pleural space and in the blood, respectively. The difference in extent of response underlines the complexity of the inflammatory processes that arise during OLV in thoracic surgery. Loco-regional inflammatory reactions were more pronounced compared to the blood. The inflammatory response occurred shortly after the surgical trauma, thus indicating that measurements must be done timely. GROα increased in the pleural fluid but neither in the BAL fluid nor in the blood and might not be useful as marker for systemic inflammatory response in complex surgery.

## List of abbreviations

OLV: One-lung ventilation; BAL: Broncho-alveolar lavage; IL: Interleukin, GROα: Growth-regulated peptide alpha; CINC-1: Cytokine-induced neutrophil chemoattractant-1; IL-1RA: Interleukin-1 receptor antagonist; ELISA: Enzyme-linked immune-sorbent assay; ARDS: Adult respiratory distress syndrome; SD: Standard deviation; IQR: Inter-quartile range; FEV1: forced expiratory volume; VC: Vital capacity; VATS: Video-assisted thoracic surgery

## Competing interests

The authors declare that they have no competing interests.

## Authors' contributions

AB sampled the fluids, performed the laboratory work and was involved in drafting the manuscript; FG performed the pleural lavages and the surgery, was involved in processing the samples and drafting the manuscript; BBS participated in the design of the study, performed the laboratory work and reviewed the draft; MT performed the BAL, was involved in processing the samples and critically reviewed the manuscript; DL performed the pleural lavages and the surgery, was involved in the statistical analysis and the drafting of the manuscript; DO was involved in the design of the study and the drafting of the manuscript and critically reviewed the paper; UZ designed the study, drafted the manuscript, performed the statistical analysis and critically reviewed the paper. All authors read and approved the final manuscript.
